# Supplementary material for: Development and Validation of a 7-eRNA Prognostic Signature for Lung Adenocarcinoma
Source: Biology (Basel). 2025 Oct 17;14(10):1431. doi: 10.3390/biology14101431 (PMC12561158; doi:10.3390/biology14101431)
Supplement: Supplementary file 1 [file biology-14-01431-s001.zip › biology-3818884-supplementary.pdf]

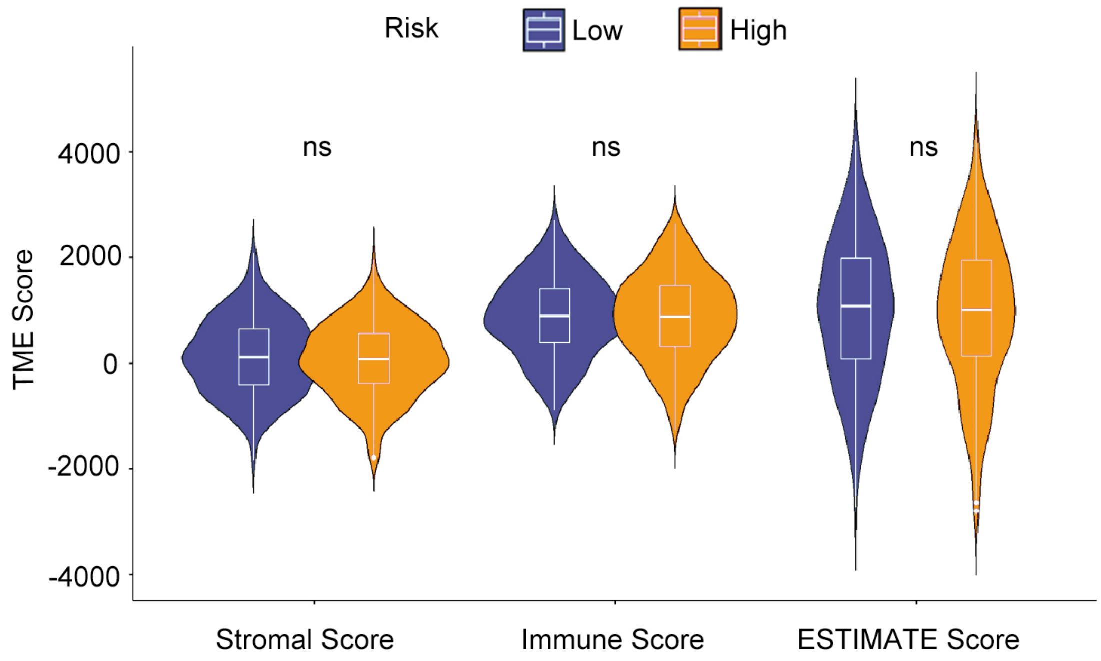

Supplementary Figure S1. Analysis of tumor immune micro environment. The ESTIMATE analysis calculated stromal cell score, immune cell score, and ESTIMATE score between high and low risk groups. ns: no significance.
